# Dynamical model parameter adjustments in model predictive filtering MR thermometry

**DOI:** 10.1186/2050-5736-3-S1-P31

**Published:** 2015-06-30

**Authors:** Henrik Odéen, Dennis Parker

**Affiliations:** 1 University of Utah, Salt Lake City, Utah, United States

## Background/introduction

In magnetic resonance guided focused ultrasound (MRgFUS) brain applications the fully insonified field-of-view (FOV) is ideally monitored. This can be achieved by k-space subsampling and using a dedicated reconstruction method, such as the previously described model predictive filtering (MPF) method.[1] MPF utilizes the Pennes Bioheat transfer equation (PBTE) and tissue thermal and acoustic properties determined from a low-power pre-treatment heating (which ideally does not deliver any thermal dose, i.e. ΔT<2°C). The accuracy of the determined tissue parameters, and hence of the MPF reconstruction, depends on the low power heating. In this work we investigate dynamical adjustment of model parameters during heating for improved MPF temperature measurement accuracy.

## Methods

All imaging used a 3D segmented EPI pulse sequence (table 1) with variable density k-space subsampling (*R=7*)[2] on a 3T MR scanner (Tim Trio, Siemens Healthcare). FUS heating was performed in a gelatin phantom with a 1MHz 256 elements phased array transducer (Imasonic/IGT). In MPF a temperature forward prediction (based on PBTE) is used in conjunction with sub-sampled k-space data to estimate the current temperatures. In this work the tissue acoustic (power density, *Q*) and thermal (conductivity, *k*) properties were determined with recently published methods[3,4] from an average of 5 low power heatings, and the MPF reconstructions were compared to fully sampled “truths.” Temperature maps were calculated with the PRF shift method. The subsampled data was reconstructed with three implementations of the MPF algorithm:

**Table 1:** MR and US parameters used for the 5 Low Power heatings (to estimate Q and k), for the fully sampled “truth,” and for the subsampled MPF heatings.

	TR/TE [ms]	Resolution [mm]	FOV [mm]	EPI	BW [Hz/px]	FA [deg]	Tacq [s]	US
Low Power Heating	22/11	1.15x1.15x2.50	147x96x45	7	752	15	4.8	3W 28.18s
Fully Sampled “Truth”	22/11	1.15x1.15x2.50	147x96x45	7	752	15	4.8	40W 28.18s
MPF	22/11	1.15x1.15x2.50	147x110x135	7	752	15	2.4	40W 28.18s

**Figure 1:**
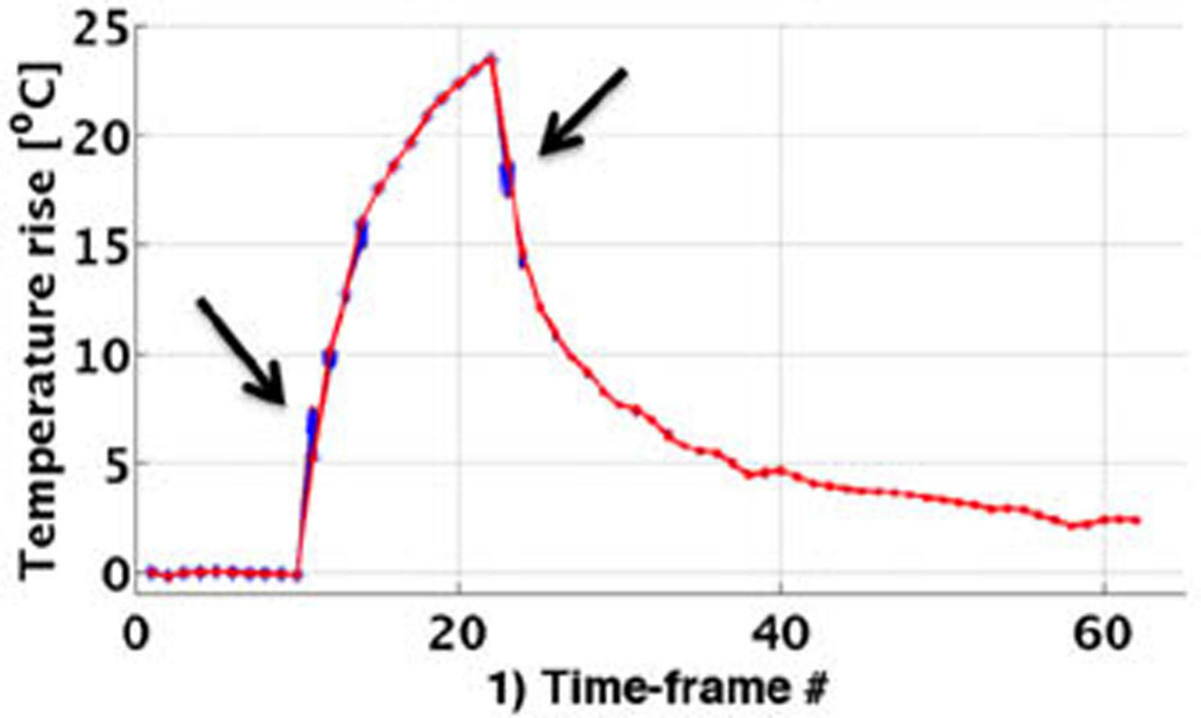
Iteratively updating MPF temperatures by adjusting Q/k. Blue dots are iterations for each dynamic time - larger adjustments are needed when US is turned on/off (black arrows). Red line indicates temperatures as obtained with the optimized parameters.

1. *No adjustment* The original implementation, using fixed values of *k* and *Q*[3,4] for all time-frames.

2. *Best current estimate* Implementation where *Q* from 1) is iteratively adjusted in each time-frame when the US is on, and k from 1) is iteratively adjusted when the US is off, so that the difference between the forward predicted model-only temperatures and the MPF estimates are minimized in each dynamic time-frame, figure 1.

3. *Final adjustment* Here the average values of *Q* and *k* achieved from all time-frames in 2) are used in the reconstruction. Since the average values of *Q/k* are used, the data cannot be reconstructed until all data is acquired, hindering real-time reconstruction.

Temperature measurement accuracy was evaluated by investigating a local (hottest voxel) and a global (all voxels with ΔT>20°C) root-mean-square-error (RMSE).

## Results and conclusions

**Figure 2:**
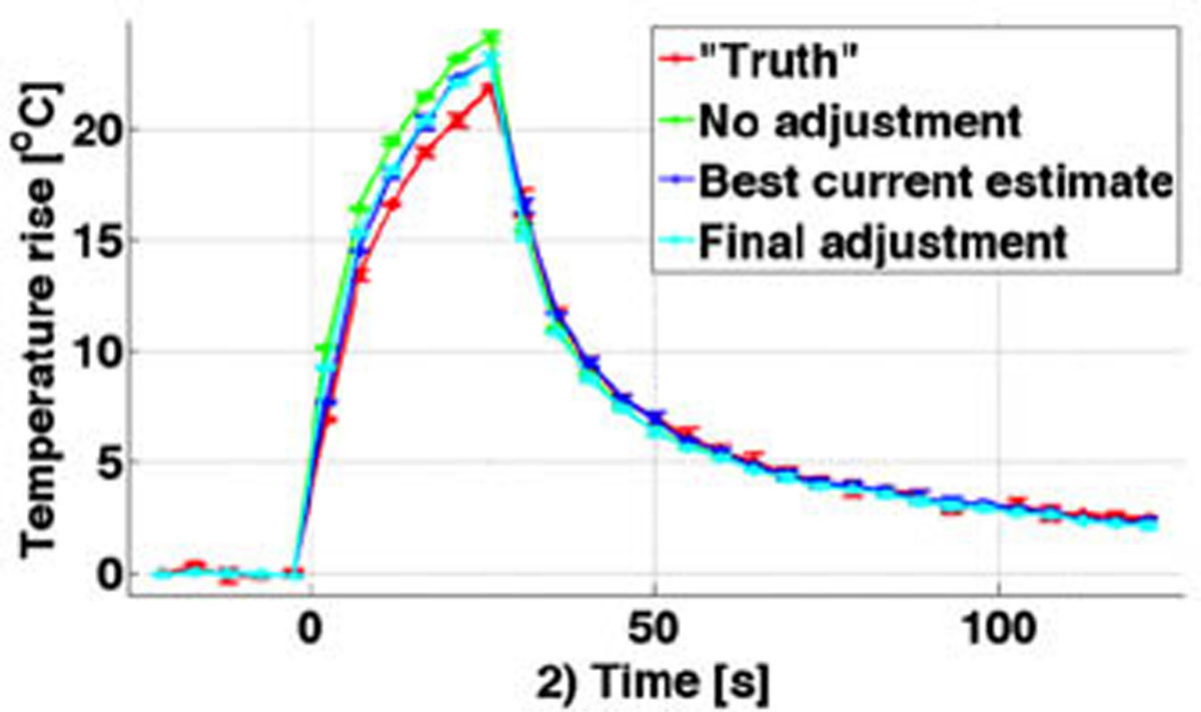
Temperature rise *vs*. time for fully sampled “truth” compared to the three implementations of the MPF algorithm. Mean and STD of three separate heatings are shown. Increased accuracy is achieved when Q/k are dynamically adjusted throughout the heating.

The mean and STD of the 5 low power heatings were 2.10±0.10°C, resulting in a negligible thermal dose (0.002 for hottest voxel). Figure 2 shows the hottest voxel *vs*. time for fully sampled “truth” and the three MPF implementations, and table 2 shows the RMSEs. A 31-50% reduction in RMSE can in the present study be achieved by dynamically adjusting *Q* and *k* during the heating. Achieving accurate estimates of tissue acoustic and thermal properties can be challenging from very low power heatings resulting in only a few degrees temperature rise. In this work we have shown that increased temperature measurement accuracy can be achieved by dynamically adjusting the model parameters as the heating progresses. Future work will aim at adjusting both *Q* and *k* during the heating by incorporating estimates of the focal spot FWHM.

**Table 2:** Mean and standard deviation (STD) of the RMSE for three repeated 40W heatings, for the three implementations of the MPF algorithm.

	RMSE	RMSE
	Hottest voxel	ΔT>20°C
1) No adjustment 2) Best current estimate 3) Final adjustment	1.37±0.03 0.69±0.03 0.94±0.04	1.89±0.25 1.26±0.31 1.24±0.25
